# Spatial and seasonal determinants of Lyme borreliosis incidence in France, 2016 to 2021

**DOI:** 10.2807/1560-7917.ES.2023.28.14.2200581

**Published:** 2023-04-06

**Authors:** Wen Fu, Camille Bonnet, Alexandra Septfons, Julie Figoni, Jonas Durand, Pascale Frey-Klett, Denis Rustand, Benoît Jaulhac, Raphaëlle Métras

**Affiliations:** 1 Sorbonne Université, INSERM, Institut Pierre Louis d’Épidémiologie et de Santé Publique, Paris, France; 2 Santé publique France, Saint-Maurice, France; 3 Laboratoire Tous Chercheurs, Université de Lorraine, INRAE, UMR 1136 Interactions Arbres-Microorganismes, Nancy, France; 4 Statistics Program, Computer, Electrical and Mathematical Sciences and Engineering Division, King Abdullah University of Science and Technology (KAUST), Thuwal, Saudi Arabia; 5 French National Reference Center for Borrelia, Hôpitaux Universitaires de Strasbourg, Strasbourg, France; 6 Institut de Bactériologie, Fédération de Médecine Translationnelle de Strasbourg, University of Strasbourg,ITI InnoVec, Strasbourg, France; 7 Centre for Mathematical Modelling of Infectious Diseases, London School of Hygiene and Tropical Medicine, London, United Kingdom; 8 Department of Infectious Disease Epidemiology, London School of Hygiene and Tropical Medicine, London, United Kingdom

**Keywords:** Lyme borreliosis, spatio-temporal epidemiology, Bayesian inference, risk factors, tick-borne diseases

## Abstract

**Background:**

Lyme borreliosis (LB) is the most widespread hard tick-borne zoonosis in the northern hemisphere. Existing studies in Europe have focused mainly on acarological risk assessment, with few investigations exploring human LB occurrence.

**Aim:**

We explored the determinants of spatial and seasonal LB variations in France from 2016 to 2021 by integrating environmental, animal, meteorological and anthropogenic factors, and then mapped seasonal LB risk predictions.

**Methods:**

We fitted 2016–19 LB national surveillance data to a two-part spatio-temporal statistical model. Spatial and temporal random effects were specified using a Besag-York-Mollie model and a seasonal model, respectively. Coefficients were estimated in a Bayesian framework using integrated nested Laplace approximation. Data from 2020–21 were used for model validation.

**Results:**

A high vegetation index (≥ 0.6) was positively associated with seasonal LB presence, while the index of deer presence (> 60%), mild soil temperature (15–22 °C), moderate air saturation deficit (1.5–5 mmHg) and higher tick bite frequency were associated with increased incidence. Prediction maps show a higher risk of LB in spring and summer (April–September), with higher incidence in parts of eastern, midwestern and south-western France.

**Conclusion:**

We present a national level spatial assessment of seasonal LB occurrence in Europe, disentangling factors associated with the presence and increased incidence of LB. Our findings yield quantitative evidence for national public health agencies to plan targeted prevention campaigns to reduce LB burden, enhance surveillance and identify further data needs. This approach can be tested in other LB endemic areas.

**Key public health message**

**What did you want to address in this study?**

Lyme borreliosis (LB) is the most common vector-borne zoonosis transmitted to humans through the bite of infected ticks in temperate regions. Understanding the impact of the environment and human exposure to tick bites on disease is important to improve surveillance and inform further field data collection.

**What have we learnt from this study?**

We found that most LB occurred in spring and summer, and in zones with more green space, habitats more suitable for deer, mild weather conditions, together with a higher chance to be bitten by a tick.

**What are the implications of your findings for public health?**

The created predictive maps provide evidence on the seasons and areas where LB occurs, allowing for enhanced surveillance, targeted prevention campaigns to further reduce the risk of tick bites and thereby LB for humans, and highlighting that more information on vectors and hosts is still needed.

## Introduction

Lyme borreliosis (LB) is a widespread zoonotic vector-borne disease in the northern hemisphere, caused by the spirochete *Borrelia burgdorferi* sensu lato (*B. burgdorferi* s.l.) species complex and transmitted by hard ticks *Ixodes* spp. [1]. LB infection is often asymptomatic or manifests as an erythema migrans (EM, typical Lyme skin rash); in rare cases, disseminated forms may occur, affecting other organs such as joints or the nervous system [1]. In western Europe, the overall estimated annual incidence of LB is ca 22 per 100,000 inhabitants, with wide variation across geographic regions, ranging from 464 per 100,000 in southern Sweden to 0.001 per 100,000 in Italy [2]. Understanding the determinants underlying the spatial heterogeneity of LB incidence in humans is necessary to better assist disease surveillance, prevention and control.

*B. burgdorferi* s.l. persistence is permitted by a complex transmission cycle at the interface between *Ixodes* spp. ticks and animal hosts [3]. *Ixodes* spp. can feed on a large variety of animals (including birds, reptiles and mammals), but only a few species can act as reservoirs for *B. burgdorferi* s.l. [3]. Furthermore, suitable vegetation habitats and climatic conditions allowing host-seeking, oviposition, eclosion, and tick molting are necessary for the completion of *Ixodes’* life cycle (egg, larvae, nymph and adult) [3]. Therefore, the presence and abundance of infected ticks depend on the distribution of specific animal hosts, but also on specific environmental and meteorological conditions. In addition, humans can increase their likelihood of exposure to infected ticks during outdoor activities.

To date, numerous studies conducted in Europe have focused on acarological risk assessment and have been realised in fragmented areas [4-11]. A large number of biotic and abiotic factors related to the ecology of *Ixodes (I.) ricinus* (the primary vector for human LB in Europe) have been tested. Among these, climatic factors such as temperature, humidity and saturation deficit have been identified as good proxies for the host-seeking activity of nymphs [4,7,8,10]. Biotic factors such as vegetation indices and rodent population distribution have been shown to be associated with tick abundance and infection rates [5,6,11,12]. Anthropogenic data characterising human outdoor activity or human exposure to infected ticks has not been well explored since such data are challenging to collect [3]. Yet, recent citizen science research has shown promising results on characterising human exposure to tick bites [13]. Exploring LB incidence within a single framework that accounts for environmental, animal, meteorological and anthropogenic factors, would be a step forward in the estimation of its spatial determinants.

Metropolitan France, located in the temperate zone of western Europe (5°W-10°E, 41°N-52°N), is affected by various climate types (e.g. semi-continental, oceanic and Mediterranean climates) and 31% of its territory is covered by forests [14,15]. LB is a growing public health concern and since 2009, the national sentinel network (Réseau Sentinelles) has monitored LB incidence [16], with increased attention nationally since 2016 [17]. To date, LB cases have been reported in all regions of the country, with a clear seasonal pattern, peaking between May and October [18]. Regional incidence rates show spatial variations, ranging from 667 cases (95% CI: 369–965) per 100,000 inhabitants in Limousin (central area) to 11 cases (95% CI: 0–33) per 100,000 inhabitants in Poitou-Charentes (western area) in 2020 [16].

In this study, we explored the determinants of spatial and seasonal LB variations in France, accounting for environmental, animal, meteorological and anthropogenic factors in a single framework. For this, we first fitted in a Bayesian framework 2016–19 LB national surveillance data to a two-part spatio-temporal statistical model to identify and estimate the spatial and seasonal determinants associated with LB presence and increased incidence. Then, we used that model to map areas and seasons at higher risk of disease, and finally, we validated our projections using data from 2020 and 2021, separately.

## Methods

### Study setting and design

Metropolitan France has 13 regions, which are further divided into administrative subdivisions of departments, with a total of 96 departments in mainland France. For our study, we divided mainland France into a grid of 1,753 cells, each with a size of ca 0.2 x 0.2 decimal degrees (dd) (~ 22 km^2^) as the unit of spatial analysis. Time was expressed in discrete units of 3 months (i.e. quarter (*Q*)), divided in winter (January to March), spring (April to June), summer (July to September) and autumn (October to December). All spatial data were rasterised and resampled to match the resolution of the grid cells and projected to the World Geodetic System 1984 (WGS84).

### Lyme borreliosis surveillance data

We produced quarterly incidence estimates for each department by aggregating departmental LB weekly incidence (per 100,000 inhabitants) from national surveillance data (https://www.sentiweb.fr) for 2016 to 2021. These incidence estimates were calculated from LB cases reported in primary care and adjusted for the number of participating general practitioners (GPs) and their participation time, the total number of licensed GPs, and the number of inhabitants in each department [19,20]. The LB case definition used was based on the guidelines of the European Study Group on Lyme Borreliosis (ESGBOR), with LB cases diagnosed by the presence of EM, or of at least one disseminated manifestation, confirmed by ELISA and Western blot tests [21].

National maps at our study resolution (0.2 x 0.2 dd) were generated from departmental quarterly incidence by spatial interpolation using ordinary kriging. The centroid of each department was used as the geographic coordinate to define the semivariogram function, i.e. the semi-variance of incidence rates from two different locations in relation to the distance between them [22]. A spherical model was used to fit the experimental semivariogram and kriged values were estimated for each grid cell [22]. We mapped the quarterly kriged LB incidence values and used this as outcome data in the model.

### Space-time model specification

*Y_ij_* represents the kriged LB incidence values at *x_i_^th^* grid cell and *t_j_^th^* quarter, which includes both zero values, indicating no reported cases, and positive continuous values, indicating the estimated incidence in that area and time period. We used a two-part model that decomposed the distribution of *Y_ij_* into a binary outcome (absence vs presence of at least an LB case) fitted to a logistic model and a continuous outcome (all positive values) fitted to a gamma model. Spatial random effects and seasonal variation were separately estimated in both models. For each part, covariates were preselected as potential risk factors according to LB eco-epidemiology, and examined independently. Selection of covariates are detailed in Table 1 and presented hereinafter.

**Table 1:** Covariates selected for the two-part model, along with descriptions and hypotheses with regards to Lyme borreliosis occurrence, France, 2016–2021

Variable	**Type**	**Description**	**Hypotheses**	**Source**
Logistic model (LB presence vs absence)^a^				
Normalised difference vegetation index (NDVI, Q)	Time-varying	NDVI values are aggregated by quarter, reflecting the photosynthetic activity of green plants.	NDVI is identified as an important proxy for predicting tick distribution, reflecting the spatial and temporal dynamics of tick activity [6]. Higher NDVI values in Q are positively correlated with the number of questing nymphs in Q, and therefore with LB presence in Q.	Copernicus Global Land Service NDVI 1 km product version 3 (January 2016 to June 2020) and NDVI 300 m product version 2 (July 2020 to December 2021) [42].
Indices of rodent species richness	Fixed-time	Predicted average number of rodent species in the field.	The number of potentially competent rodent species may influence the spread of the vector-borne pathogens [43].	Layer containing five rodent species, namely *Apodemus agrarius, Apodemus flavicollis, Apodemus sylvaticus, Microtus arvalis, Clethrionomys glareolus* [43].
Gamma model (Increase of LB incidence conditional on LB presence)^b^				
Index of deer presence	Fixed-time	Percentage (%) of suitable habitat for deer.	High deer density is associated with increased tick abundance, suggesting a higher risk of LB [35].	Presence layers of roe deer and red deer [44,45].
Soil temperature (ST, Q)	Time-varying	Average daily maximum soil temperature (°C, level 1:0–7 cm), averaged by quarter.	ST mainly influence tick development. We assumed that ST in Q favours tick’ questing activity, which relates to increased human LB in Q. The temperature thresholds for questing activity (average maximum ST) that have been observed is 10 °C for larvae and 7–8 °C for nymphs, with peaks occurring at 15–17 °C [7,10].	ECMWF Reanalysis v5 - Land dataset (2016–21) [40].
Saturation deficit (SD, Q)	Time-varying	Index calculated from average daily mean air temperature and humidity, averaged by quarter.	SD could explain important variations in numbers of questing ticks, with peaks being observed between 2 and 7 mmHg, and initiating a decline when SD > 5 mmHg [8]. Decreasing SD values in Q is assumed to be positively associated with disease incidence in that same quarter.	SD was calculated using air temperature (AT) and relative humidity (RH) from the ECMWF Reanalysis v5 dataset (2016–2021) [41], using the formula: SD = (1-RH/100) *4.9463*exp (0.0621*AT) [10].
Rainless days (Q)	Time-varying	Cumulative number of rainless days per quarter. A rainless day is defined as a daytime period (06.00–18.00) when the average depth of precipitation covering the entire surface is less than 1 mm per hour.	Precipitation is assumed to be negatively correlated with human activity outdoors [46]. We assumed that more rainless days in Q is associated with humans being outdoors in Q and is therefore expected to be positively associated with an increased LB incidence in Q.	ECMWF Reanalysis v5 dataset (2016–21) [41].
Frequency of tick bite reports (Q)	Time-varying	Proportion of quarterly tick bite reports per department, adjusted for weighted population at risk.	People may develop an LB infection after being bitten by ticks [47]. The number of tick bites in Q is assumed to be positively correlated with LB incidence in that same quarter.	Information collected by Signalement TIQUE smartphone application and website (July 2017 to December 2021) [48].

AT: air temperature; ECMWF: the European Centre for Medium-Range Weather Forecasts; LB: Lyme borreliosis; NDVI: normalised difference vegetation index; Q: quarter; RH: relative humidity; SD: saturation deficit; ST: soil temperature.

^a^ Environmental and *Borrelia* reservoir factors associated with infected ticks correlate with the spatiotemporal presence of LB cases in humans.

^b^ Animal host, meteorological and anthropogenic factors influence tick density, questing activity and the frequency of human–tick contact, associated with LB incidence.

The complete model is defined as follows:

LogitProbYij>0= αxi,tjTVxi,tj+ξbxi+ωb(tj) Elog⁡Yij|Yij>0= βxi,tjTGxi,tj+ξgxi+ωg(tj) 

Where *V* and *G* are vectors of covariates at location *x_i_* at quarter *t_j_* associated with the outcome in binary part and continuous part, respectively. *⍺* and *β* are vectors of coefficients for the covariates and we assigned a Gaussian prior with mean of zero and precision of 0.001. The spatial random effects, for the logistic *ξ_b_(x_i_),* and gamma *ξ_g_(x_i_)* models were specified using a Besag-York-Mollie (BYM) model. It consists of an intrinsic conditional autoregressive model for spatially structured effects *u_i_*, and an independent identically distributed Gaussian model for spatially unstructured effects *𝜈_i_* [23].

$u_{i}|u_{h},\;i\neq h,\;\tau_{1}∼Normal(\frac{1}{n_{i}}\sum_{i∼h}u_{h},\frac{1}{n_{i}\tau_{1}})$

$v_{i}∼Normal(0,(\frac{1}{\tau_{2}}{)}^{2})$

where *i~h* indicates that two locations *i* and *h* are first-order neighbours, *n_i_* is the number of neighbours of location *i*, with 𝜏_1_ and 𝜏_2_ being the precision parameters.

Seasonal variations for the logistic *ω_b_(t_j_)*, and gamma *ω_g_(t_j_)* parts can be represented as a set of random vectors 𝝎 = (ω_1_, ω_2,_…, ω_j_) with periodicity *s* . The density for 𝝎 is derived from the *j-s + 1* increments as

$\pi (\omega |\;\tau_{3})\;\propto \;{\tau_{3}}^{\frac{\left(j-s+1\right)}{2}}exp\left\{-\frac{\;\tau }{2}\sum (\omega_{t}+\omega_{t+1}+\ldots +\omega_{t+s-1}{)}^{2}\right\}$

Here *s* = 4 denotes the number of quarters in 1 year and *j* = 24 denotes the total number of quarters in 2016–21. We set the priors for precision parameters 𝜏_1_, 𝜏_2_, 𝜏_3_ as Gamma (1, 0.00005).

### Inference framework and parameter estimation

The fixed and random effects coefficients were estimated from data by Bayesian inference. We used the integrated nested Laplace approximation (INLA) method to approximate the posterior marginal distribution of the parameters of the two-part model. All analyses were performed in R version 4.0.5 using the INLA package [24].

### Model selection and validation

Pearson correlation analysis was first used to test the correlation between candidate covariates, and only these with a coefficient less than 0.7 were retained for the model. In addition to those listed in Table 1, candidate covariates excluded from the model were soil temperature in the previous year, seasonal air temperature and humidity, forest type, daylight hours and Google trends data related to LB. Univariable and multivariable analyses were then conducted, and covariates were categorised by biological relevance or quartiles. We used the widely applicable information criterion (WAIC) to select the preferred multivariable model [25]. Cross-validation was performed using data from 2020 and 2021, separately. For all study years, maps of the probability of LB case presence, the mean predicted incidence value and their standard deviation were produced for each season. The empirical distributions of the probability integral transform (PIT) were plotted to assess the predictive performance of our model [26]. Finally, the overall annual national incidence with their 95% confidence interval were calculated and compared with the Réseau Sentinelles values [27].

### Environmental, animal and meteorological data

We calculated NDVI average values for each grid cell by quarter. For the animal host data, we calculated the percentage of deer habitat and the average number of rodent species in each grid cell, fixed in time.

Soil temperature (ST), air temperature (AT) and relative humidity (RH) data were summarised quarterly as maximum and mean averages and resampled to each grid cell. Precipitation (PP) was used to calculate the number of rainless days per quarter. All processing of meteorological data was performed using raster, ncdf4, ecmwfr and keyring packages in R version 4.0.5 [28].

### Anthropogenic data on tick bites

A total of 43,915 human tick bite reports were included in the analysis. For each report, we extracted the information on reporting dates and GPS position (WGS84). Since information before July 2017 was not available, we made a compromise by retaining the average seasonal variation for each department throughout 2016–19 in the model. Data for 2020 and 2021 were used separately for the forecast of each year (see Supplementary Material S1 for the calculation of frequency of tick bite reports per department and quarter).

## Results

In Kriging interpolation, the semi-variogram shows that the incidence distribution displayed spatial autocorrelation up to 110 km (see Supplementary Material S2 for the spatial interpolation by ordinary kriging methods and Figure S1 for the results of fitted spherical model from 2016–19). Smoothed maps of quarterly kriged LB incidence for the study period were used as our outcome variable, exhibiting an increased incidence in spring and summer, mainly in the north-eastern and eastern France. The maps can be found in Supplementary Figure S2.

### Identified risk factors from the two-part model

The results of the preferred two-part model with the smallest WAIC value are reported in Table 2, and detailed as follows. The results of the first part of the model (logistic model), show that areas with higher vegetation activity, i.e. NDVI values above and equal to 0.6, had a 37% higher odds of LB presence than areas with lower NDVI (OR: 1.37, 95% credible interval (Crl): 1.25–1.51). Whereas the rodent species richness index was negatively correlated with LB presence, with the odds of LB occurring being 17% lower in areas with more species (OR: 0.83, 95% Crl: 0.70–0.99). In the second part of the model (gamma model), areas with more than 60% and 80% cover of suitable deer habitat had 1.13 and 1.25 times higher risk of LB compared with areas with less than 40% cover (relative risk (RR):  1.13, 95% Crl: 1.03–1.24 and RR: 1.25, 95% Crl: 1.11–1.41, respectively). Also, the risk of LB in quarter Q increased by 1.18 in areas with mild soil temperature (ST: > 15–22 °C, RR: 1.18, 95% Crl: 1.11–1.25) in the quarter, but decreased for temperatures higher than 22 °C (ST: > 22 °C, RR: 0.76, 95% Crl: 0.69–0.82). In addition, the risk of LB increased by 1.08 and 1.18 in areas with saturation deficit (SD) values ranging between 1.5–3 mmHg and 3–5 mmHg compared with areas with SD below 1.5 mmHg (RR: 1.08, 95% Crl: 1.02–1.15 and RR: 1.18, 95% Crl: 1.05–1.31, respectively). Finally, the frequency of tick bite reports was also an important predictor of LB risk, with an increased incidence of LB in locations and seasons where a higher proportion of bites were reported (Table 2).

**Table 2:** **Two-part multivariate model disentangling covariates associated with the presence and increased incidence of Lyme borreliosis, France, 2016**–**2019**

Variable	Unit/category	Risk coefficients	95% Crl^a^
**Logistic model (LB presence vs absence)** ^b^			
NDVI (Q)	< 0.6	Ref.	
	≥ 0.6	1.37	1.25–1.51
Rodent species richness	> 1 to ≤ 3.27	Ref.	
	> 3.27 to ≤ 5	0.83	0.70–0.99
**Gamma model (Increase of LB incidence conditional of LB presence)** ^c^			
Index of deer presence	≤ 40%	Ref.	
	> 40 to ≤ 60%	1.04	0.97–1.12
	> 60 to ≤ 80%	1.13	1.03–1.24
	> 80%	1.25	1.11–1.41
ST (Q)	≤ 7 °C	Ref.	
	> 7 to ≤ 15 °C	1.04	0.97–1.10
	> 15 to ≤ 22 °C	1.18	1.11–1.25
	> 22 °C	0.76	0.69–0.82
SD (Q)	≤ 1.5 mmHg	Ref.	
	> 1.5 to ≤ 3 mmHg	1.08	1.02–1.15
	> 3 to ≤ 5 mmHg	1.18	1.05–1.31
	> 5 mmHg	1.06	0.94–1.20
Frequency of tick bite reports (Q)	≤ 0.05%	Ref.	
	> 0.05 to ≤ 0.25%	1.20	1.14–1.27
	> 0.25%	1.35	1.24–1.46

Crl: credible interval; LB: Lyme borreliosis; NDVI: normalised difference vegetation index; OR: odds ratios; Q: quarter; RR: relative risk; SD: saturation deficit; ST: soil temperature.

^a^ The risk coefficients (odds ratio and relative risk, respectively for the logistic and gamma model) and their 95% Crl were obtained by exponentiating the coefficients’ mean value of the posterior marginal distribution and their 95% Crl.

^b^ The risk coefficient refers to the odds ratio in the logistic model, representing the ratio of the odds of an LB case occurring in the current category compared to the odds of an LB case occurring in the reference category.

^c^ The risk coefficient refers to the relative risk in the gamma model, representing the ratio of the probability of an LB case occurring in the current category compared to the reference category.

### Spatial and seasonal maps

**Figure 1:**
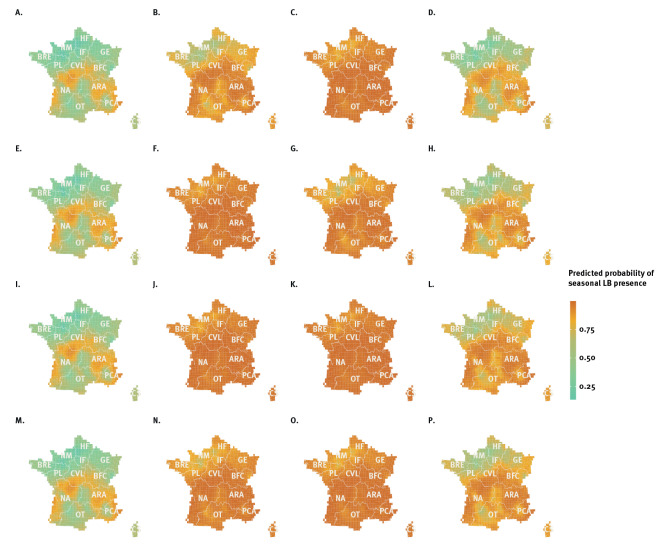
Predicted probability of seasonal Lyme borreliosis presence, France, 2016–2019

**Figure 2:**
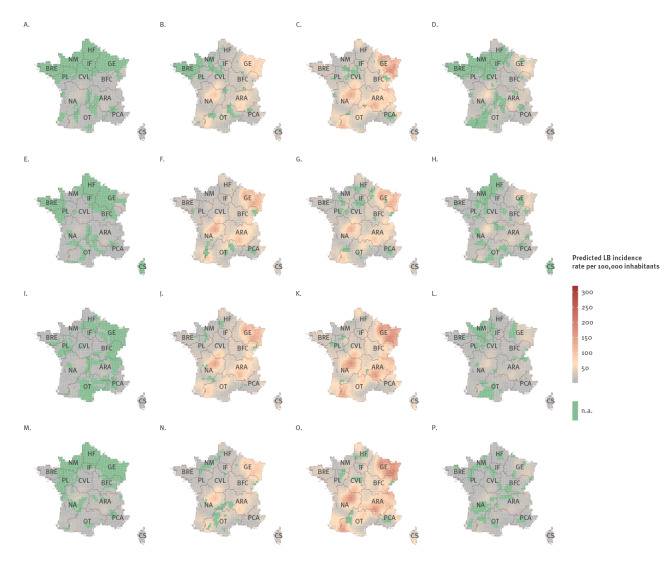
Predicted seasonal Lyme borreliosis incidence rate per 100,000 inhabitants, France, 2016–2019

To visualise our results, we generated projection maps for each part of the model (Figure 1 and Figure 2). Figure 1 shows the predicted quarterly probability of LB presence. We observed a seasonal pattern of disease occurrence, with higher probabilities in spring and summer, in almost every region of the country. Conversely, the probability of occurrence was much lower in autumn and winter but revealed important geographic heterogeneity, with high-risk areas concentrated in Grand Est (GE), Bourgogne-Franche-Comté (BFC), and Auvergne-Rhône-Alpes (ARA) regions in eastern France, and in the Nouvelle Aquitaine (NA) and Occitanie (OT) regions in midwestern and south-western France.

LB: Lyme borreliosis.

Panels A–D show 2016 predicted values, E–H show 2017 predicted values, I–L show 2018 predicted values and M–P show 2019 predicted values. Each column from left to right indicates the winter (January to March), spring (April to June), summer (July to September) and autumn (October to December) of each year. The darker orange colour indicates a higher probability of LB presence, while the lighter green indicates a lower probability. A French shapefile with a resolution of ca 0.2 x 0.2 decimal degrees (~ 22 km^2^) was used in this study (See Supplementary Source 1 for the French shapefile template).

Each of the 13 regions in mainland France is indicated on the maps by initials (HA: Hauts-de-France; NM: Normandie; IF: Île-de-France; GE: Grand Est; BRE: Bretagne; PL: Pays de la Loire; CVL: Centre-Val de Loire; BFC: Bourgogne-Franche-Comté; NA: Nouvelle Aquitaine; ARA: Auvergne-Rhône-Alpes; OT: Occitanie; PCA: Provence-Alpes-Côte d’Azur; CS: Corse).

LB: Lyme borreliosis; n.a.: not included in the gamma model.

Panels A–D show 2016 predicted incidence, E–H show 2017 predicted incidence, I–L show 2018 predicted incidence and M–P show 2019 predicted incidence. Each column from left to right indicates the winter (January to March), spring (April to June), summer (July to September), and autumn (October to December) of each year. The darker red areas indicate a higher incidence of LB, while green areas indicate no LB cases reported (i.e. observations equal to zero that are excluded from the gamma model). A French shapefile with a resolution of ca 0.2 x 0.2 decimal degrees (~ 22 km^2^) was used in this study (See Supplementary Source 1 for the French shapefile template).

Each of the 13 regions in mainland France is indicated on the maps by initials (HA: Hauts-de-France; NM: Normandie; IF: Île-de-France; GE: Grand Est; BRE: Bretagne; PL: Pays de la Loire; CVL: Centre-Val de Loire; BFC: Bourgogne-Franche-Comté; NA: Nouvelle Aquitaine; ARA: Auvergne-Rhône-Alpes; OT: Occitanie; PCA: Provence-Alpes-Côte d’Azur; CS: Corse).

Seasonal LB incidence rates predicted for 2016 to 2019 are presented in Figure 2. They exhibit geographic heterogeneity, seasonality and interannual variations. Over the 4 years, higher incidence rates were observed in the spring and summer in Grand Est (GE), Auvergne-Rhône-Alpes (ARA) and Nouvelle-Aquitaine (NA) regions, consistent with the results of the logistic model. The spring maps for 2017 and 2018 show a similar pattern (Figure 2F and J), with slightly higher predicted values than in 2016 and 2019 (Figure 2B and N). The summer patterns displayed increased incidence in 2018 and 2019 in GE, ARA and NA regions (Figure 2K and O), compared with 2016 and 2017 (Figure 2C and G). The average incidence in spring over the 4 years was 35 cases per 100,000 inhabitants, and the grid cell with the maximum incidence value reached 159 per 100,000 inhabitants. Similarly, for 2016–19 summers, the average incidence was 51 per 100,000 inhabitants, while the maximum summer value reached 212 per 100,000 inhabitants.

**Figure 3:**
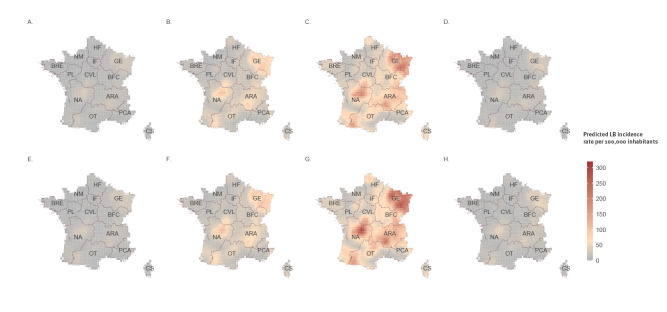
Predicted seasonal Lyme borreliosis incidence rate per 100,000 inhabitants, France, 2020–2021

Using the model informed with 2016–19 data, we produced the forecast maps for 2020 and 2021, as well as corresponding standard deviation maps, which are shown in Supplementary Figure S7. The results of the logistic model showed a similar spatial and seasonal pattern, aligning with previous years, shown in Supplementary Figure S6. The results of the gamma model are shown in Figure 3. The overall spatial and seasonal patterns for 2020 and 2021 are comparable to those of previous years. The incidence in 2020 (with the summer maximum value reaching 229/100,000 inhabitants) was almost equivalent to that in 2019 (Figure 3A to D), whereas in the following year 2021, LB incidence was predicted to increase in a subset of grids in GE, ARA and NA regions, with a maximum predicted LB incidence of 307 per100,000 inhabitants in summer (Figure 3G). In addition, the annual predicted mean incidence rates and their 95% CI, calculated at national level for the entire period (2016–21), suggest that the incidence of LB in France is broadly stable and show a similar trend to Réseau Sentinelles’ values (See Supplementary Figure S10 for the graph of comparison of the overall national predicted and observed annual LB incidence, France, 2016–21). The PIT histograms for 2020 and 2021 are satisfying and do not show an over or under-dispersed predictive distribution, suggesting a well-calibrated predictive performance (See Supplementary Figure S8 for the histogram of the probability integral transform (PIT), 2020 and Figure S9 for the histogram of the probability integral transform (PIT), 2021). The other tested models are presented in Supplementary Table S1. The number of rainless days was ultimately not included in the final model because not improving our model fit.

LB: Lyme borreliosis.

Panels A–D show 2020 predicted incidence, E–H show 2021 predicted incidence. Each column indicates in turn the winter (January to March), spring (April to June), summer (July to September), and autumn (October to December) of each year. The darker red areas indicate those with a higher incidence, while grey areas indicate those with low LB incidence. A French shapefile with a resolution of ca 0.2 x 0.2 decimal degrees (~ 22 km^2^) was used in this study (See Supplementary Source 1 for the French shapefile template).

Each of the 13 regions in mainland France is indicated on the maps by initials (HA: Hauts-de-France; NM: Normandie; IF: Île-de-France; GE: Grand Est; BRE: Bretagne; PL: Pays de la Loire; CVL: Centre-Val de Loire; BFC: Bourgogne-Franche-Comté; NA: Nouvelle Aquitaine; ARA: Auvergne-Rhône-Alpes; OT: Occitanie; PCA: Provence-Alpes-Côte d’Azur; CS: Corse).

## Discussion

We present a national-level spatial assessment of seasonal LB occurrence in Europe, allowing the disentanglement of factors associated with LB presence and increased incidence in mainland France. Our results provide evidence that seasonal LB presence was positively associated with higher vegetation index, which was used as a proxy for the presence of *Ixodes*, while increased incidence was associated with a higher index of deer presence, mild seasonal soil temperature, moderate air saturation deficit and higher tick bite frequency. This model illustrates that the use of surveillance data, combining environmental, animal host, meteorological and human tick bite reports into a unified analytical framework, can be used to understand the seasonal and spatial patterns of LB occurrence. This approach can be tested in other areas where surveillance data and human tick bite information are available (such as Switzerland, the Netherlands and Belgium) [29,30] and, if the model results show similarities across several different pilot regions, could also provide insights into LB burden estimation across continental Europe.

One strength of our study lies in the use of kriging interpolation to obtain a continuous process of LB distribution in mainland France, allowing the inclusion of climatic and environmental variables at a fine resolution [22]. In addition, our Bayesian BYM two-part model allowed for the simultaneous consideration of the semi-continuous outcome variable, and for the spatial correlation and seasonal variations, by reducing the structural and non-structural random effects on parameter inference [23]. Predictions and validations using 2020 and 2021 data allowed the identification of similar spatial and seasonal patterns, giving confidence in the robustness of our model. During the COVID-19 pandemic in 2020, two national lockdowns were implemented in France. The first lockdown (17 March–11 May 2020) only allowed movements within 1 km of the residence (for outdoor physical activities, walks) and occurred before the peak of reported LB cases [31]. Then, the second lockdown (30 October–15 December 2020) imposed similar restrictions on outdoor recreational activities, followed by a nationwide curfew that no longer restricted movement during daytime [32]. Therefore, we assumed the COVID-19 lockdowns did not affect the overall spatial and seasonal patterns of human encounters with ticks throughout 2020. In addition, in 2020, 87.6% of LB cases reported their tick-bite in their department of residence, consistent with previous years (84.4–88.8% in 2016–19) [33]. For prediction and model validation, the quarterly proportions of human tick bite frequency were processed for 2020 and 2021 separately at the departmental level, displaying similar spatial patterns compared with previous years. Finally, even if we could not evaluate the care-seeking patterns of individuals (access to GP’s and care facilities) during the 2020 lockdown period that could have had an effect on LB notifications, our predictions remain in line with previous years.

Our prediction maps highlight the seasonal pattern of LB occurrence, i.e. spring and summer, and revealed the heterogeneous distribution of LB across and within regions in mainland France. Areas with a higher LB incidence burden were located in eastern (GE, BFC, and ARA), midwestern (NA), and south-western (OT) France, in agreement with previous studies [18]. The prediction map for the summer 2021 showed an increased incidence in some grid cells in high-risk areas. This can be explained by the fact that abundant rainfall in summer 2021 resulted in lower quarterly saturation deficit (SD) values than in previous years (Supplementary Figure S4), with values in the 3–5 mmHg category being associated with increased incidence. Moderate saturation deficits might favour ticks’ questing activity, hence contributing to an increase in our predicted incidences but in fact did not necessarily increase the tick infection rates with *Borrelia* or, conversely, could have led to a reduced risk of tick bites if human outdoor activity had lessened. Yet, when aggregated at the national level, the incidence of LB in France remained rather stable from 2016 to 2021.

The value of NDVI data has been discussed in earlier studies as an important predictor of tick-suitable habitats and questing nymph abundance [6,11]. We incorporated NDVI into the model and found that areas and seasons with higher NDVI values were positively correlated with LB presence, consistent with the results of acarological studies [11]. In addition, we introduced an index of rodent species richness in the logistic model and found a weak negative correlation between LB presence and a greater number of rodent species. There are several possible explanations for this. First, the available information provides only the number of rodent species at each site, the exact species and density variation is unknown. More competent species at low densities may not be able to maintain the local intensity of pathogen transmission [34]. Second, the presence of more rodent species also suggests that the local environment is friendly to small rodents and their predators and that coexistence of other incompetent reservoir hosts may have a diluting effect on tick infection rates [34]. If larvae attach to these incompetent hosts, nymphal infection rates are instead reduced. For further insight, a nationwide field survey of species associated with *Borrelia* reservoirs and their densities would be necessary, as well as rates of *Borrelia* infection in ticks in several regions.

In addition, we found that higher LB incidence was associated with deer-suitable habitat coverage of more than 60% of each grid area. The percentage of deer habitat reflects the likelihood of deer presence and their abundance. Roe deer and red deer are known to be the primary productive hosts for adult female ticks and can maintain high tick populations locally [35]. As incompetent *Borrelia* reservoir hosts, some studies hypothesised that ticks lose *Borrelia* infection after feeding on deer [36]. However, others have suggested that an increase in overall tick density leads to an increase in the density of infected nymphs, resulting in increased LB risk [37]. A long-term study in Norway also showed that high spatial and temporal densities of deer lead to an increased incidence of LB in humans, supporting our results [35].

Our findings also point to a positive correlation between areas with higher LB incidence and mild soil temperature (15–22 °C) and moderate saturation deficit (1.5–5 mmHg) during the peak season of cases’ occurrence. Optimal ground and air temperatures (at 60 cm) for tick activity have been shown to be between 13 and 23 °C, and most questing tick activity was found to occur between 2 and 7 mmHg, consistent with our model results [8-10]. Thus, we speculate that tick development of any stage is favoured during mild springs, and humans, as accidental hosts, are exposed to tick bites (nymphal and adult stages) during the questing phase in spring and summer seasons, resulting in an elevated risk of LB. In addition, the higher frequency of human outdoor activity during the summer months, which overlaps with tick habitat and questing activity could also contribute to increased incidence.

The use of citizen-based health data is growing in interest in epidemiological research and has recently been applied to tick bite tracking in several countries [13,29,30]. These citizen engagement-based data can be considered complementary to surveillance data, by offering the potential to get more accurate information on tick bite exposure. Here, CiTIQUE data allowed us to account for the spatial and temporal variations in the risk of human exposure to tick bites. CiTIQUE data was assumed to capture both human outdoor activity and tick encounters, largely outperforming the number of rainless days (variable initially used as a surrogate to human outdoor activities) in improving model fit, and highlighting the importance of citizen-based research initiatives.

There are some limitations to consider. Firstly, LB surveillance relies on voluntary reporting of cases by sentinel general practitioners (SGPs) in the Reseau Sentinelles. The national LB surveillance incidence rates used in this study were estimated from cases reported by SGPs in each department. To minimise the reporting bias, the heterogeneous distribution, number and participation of SGPs were adjusted to calculate incidence rates [20]. However, the data have been collected in a consistent manner since 2009 and the spatial and seasonal patterns have shown stability across years, and are consistent with hospital-based data for LB [18], corroborating the good quality of the dataset. Secondly, the data collection from the national CiTIQUE programme is primarily targeted at people who are interested in ticks and have a smartphone. Uneven public awareness of ticks and differences in the frequency of Signalement TIQUE application use across departments may affect tick bite reporting. Considering that human recreational activities are closely related to the probability of tick bite exposure, we adjusted the population at risk by leisure-related green space for each department. Moreover, since CiTIQUE data was available only after July 2017, we calculated seasonal averages using 2017–19 data and used these estimates for the whole study period 2016–19. By doing so, we assumed that exposure to tick bite had seasonal variations, but was similar from one year to another. However, it was shown in the health survey (Baromètre Santé) conducted by Santé publique France that public awareness of tick bites and LB increased in 2019 compared with 2016, suggesting that using seasonal averages would potentially bias the input data used for the model [38]. Thirdly, the zero values in the two-part model (estimated by kriging) are considered to be true zeros. We observed that most zero values occurred in winter and autumn, consistent with the epidemiological characteristics of LB occurrence, but zero values were also generated in spring and summer in some high-risk areas. Therefore, zero values in our data could be a result of the absence of LB cases or under-reporting. This also emphasises the need for enhanced surveillance. Fourthly, both animal-related variables used in this study are predictive indexes that combine observed animal presence data, land cover data, and annual climate variables aggregated over multiple years, and vary only spatially, as distinguished from genuine data on host abundance. Seasonal variation in deer and rodent populations would provide more realistic assumptions for predicting the density of infected ticks during the same season or the one following, yet this information is difficult to obtain at a national scale. Finally, in our analysis, the effects of meteorological factors were assumed to have an impact on tick development and questing activity. Yet, we acknowledge that they also affect the reproduction rate and activity of other potential hosts (especially small mammals), as well as their food resources (e.g. tree seeds) [39]. Meteorological factors may also be associated with human propensity to be outdoors, particularly when weather favours outdoor recreational activities and overlaps with weekends, holidays, and summer vacations, potentially generating more reports of human tick bites. Given that some risk factors have now been identified, further exploration of the complexity and dynamics of human LB incidence should be complemented by using mathematical models.

## Conclusion

We present a national-level spatial assessment of seasonal LB occurrence in Europe, that disentangles factors associated with the presence and increased incidence of LB. Our model results showed that a higher vegetation index in spring and summer was correlated with the presence of LB, while increased incidence was associated with higher indexes of deer presence, moderate soil temperature and saturation deficit and higher tick bite frequency. Our findings yield quantitative evidence for national public health agencies to plan targeted prevention campaigns to reduce LB burden and enhance surveillance, as well as highlighting the need for further data collection on vectors and reservoir hosts. This approach can be tested in other LB endemic areas, and revised when further data becomes available. In addition, we highlight key factors such as soil temperature, deficit saturation, and frequency of human tick bites that need to be explored when using mathematical models to further investigate the complexity of LB transmission dynamics.

## Supplementary Data

987000 Supplement
