# Supplementary material for: Spatial and seasonal determinants of Lyme borreliosis incidence in France, 2016 to 2021
Source: Euro Surveill. 2023 Apr 6;28(14):2200581. doi: 10.2807/1560-7917.ES.2023.28.14.2200581 (PMC10283463; doi:10.2807/1560-7917.ES.2023.28.14.2200581)
Supplement: Supplement [file 22-00581_FU_SUPPLEMENT.pdf]

Supplementary Material

This supplementary material is hosted by *Eurosurveillance* as supporting information alongside the article [Spatial and seasonal determinants of Lyme borreliosis incidence in France, 2016 to 2021], on behalf of the authors, who remain responsible for the accuracy and appropriateness of the content. The same standards for ethics, copyright, attributions and permissions as for the article apply. Supplements are not edited by *Eurosurveillance* and the journal is not responsible for the maintenance of any links or email addresses provided therein.

Table of contents

Supplementary Material S1 Calculation of the frequency of tick bite reports per department and per quarter ...2

Supplementary Material S2 Spatial interpolation by ordinary kriging method 3

- Figure S1. Results of fitted spherical model from 2016–2019. 3
- Source 1 French shapefile template. 3
- Figure S2. Smoothed maps of quarterly kriged Lyme borreliosis incidence, France, 2016–2019 4
- Figure S3. Smoothed maps of quarterly kriged Lyme borreliosis, France, 2020–2021. 4

Supplementary Material S3 Description of meteorological covariates 5

- Figure S4. Quarterly averaged daily mean saturation deficit (SD), France, 2016–2021. 5
- Figure S5. Quarterly averaged daily maximum soil temperature (ST), France, 2016–2021. 6

Supplementary Material S4 Prediction results of the logistic model....................7

- Figure S6. Predicted probability of seasonal Lyme borreliosis presence, France, 2020–2021 7
- Figure S7. Standard deviation of predicted mean incidence, France, 2016–2021. 8
- Table S1 Results of other tested multivariable models. 9

Supplementary Material S5 Cross-validation of the gamma model 10

- Figure S8. Histogram of the probability integral transform (PIT), 2020. 10
- Figure S9. Histogram of the probability integral transform (PIT), 2021. 10
- Figure S10. Comparison of the overall national predicted and observed annual LB incidence, France, 2016–2021. 11

References 12

Supplementary Material S1 Calculation of the frequency of tick bite reports per department and per quarter

A total of 43,915 human tick bite reports were included in the analysis which was collected during 2017-2021 by the national CiTIQUE project (www.citique.fr) [1,2]. For each report, we extracted the information on reporting dates and GPS position (WGS84). Since the tick species in each report was not available, we used all tick bites reports. However, 96% of a random sample of 2009 of those human-attached ticks (at least 150 randomly selected from each region of France) were identified morphologically and genetically as *Ixodes ricinus*. This gives weight to our assumption that *Ixodes ricinus* was the main tick species responsible for the large majority of bites in the dataset.

We calculated the departmental and quarterly proportion of tick bite reports. For the period 2016-2019, we produced averaged quarterly values using the data available (i.e., from July 2017), and used these for each year of the period. For the year 2020 and 2021, we used corresponding 2020 and 2021 CiTiQUE data. Considering that human recreational activities are closely related to the probability of tick bite exposure, we assumed that the population at risk is proportional to the fraction of leisure-related green space in the department.

$$N_h(d,q) = \frac{N(d,q)}{10^{-5} H_d \, G_d} \quad (1)$$

$$N_h = \sum_{d=1}^{D} \sum_{q=1}^{Q} N_h(d,q) \quad (2)$$

$$P(d,q) = \frac{N_h(d,q)}{N_h} \quad (3)$$

where d=1, 2, …, D for each department, and q=1, 2, …, Q for each quarter. Here $Q = 4$, we used the available data during 2017–19 to calculate the quarterly average for four quarters (in order January to March (winter), April to June (spring), July to September (summer), October to December (autumn)). H_d is the number of inhabitants in department d, G_d represents the cumulative percentages of recreational areas in that department where people are likely to encounter tick bites. They are derived from the Corine land cover dataset (CLC 2018), and selected land types are listed below: 311 Broad-leaved forest 312 Coniferous forest 313 Mixed forest 324 Transitional woodland-shrub 322 Moors and heathland 321 Natural grasslands; 243 Land principally occupied by agriculture, with significant areas of natural vegetation 231 Pastures; 141 Green urban areas 142 Sport and leisure facilities [3]. $N(d,q)$ is the average number of tick bites reported in department d and in quarter q. In equation (1), we adjusted the population weight of department d to obtain the number of quarterly reported tick bites per 100,000 inhabitants, denoted as $N_h(d,q)$. In equation (2) the population-adjusted tick bite reports N_h was aggregated for all department and all quarters. We then calculated the quarterly proportion of tick bite reports $P(d,q)$ for each department. To be consistent with the size of the grid cells analysed, we rasterized the department polygons and assigned the same value $P(d,q)$ to grid cells belonging to the same department. All operations were performed using the raster library in R version 4.0.5 [4].

Population data were obtained from the 2017 national census by the National Institute of Statistics and Economic Studies (INSEE) [5]. French administrative boundaries shapefiles were downloaded from the Global Administrative Areas (GADM) Data [6]. Geographical information was retrieved from the National Institute of Geography and Forestry (IGN) [7].

Supplementary Material S2: Spatial interpolation by ordinary kriging method

To apply the ordinary kriging method, we assumed that the difference of LB incidences between two locations depends only on their distance [8]. We first estimated the semivariance function, which is defined by the following equation:

$$\hat{\gamma}(h) = \frac{1}{2N(h)} \sum_{i=1}^{N(h)} \big(Z(x_i + h, y_i + h) - Z(x_i, y_i)\big)^2$$

where Z (x_i, y_i) represent the observed LB incidence value at coordinates (x_i, y_i) and $N(h)$ is the number of pairs of observations at a distance h from each other. The computation of $\hat{\gamma}(h)$ is repeated sequentially for 2h, 3h, ... , kh, By default, k is set up to 10 and all operations were performed using the kriging library in R version 4.0.5.[4] We then fitted the observed semivariogram with a spherical model and estimated the parameters of the theoretical model.

$$\begin{cases} \gamma(h) = 0 & for\ h = 0 \\ \gamma(h) = C_0 + C_1 \left\{ \frac{3}{2}\frac{h}{C_2} - \frac{1}{2}\left(\frac{h}{C_2}\right)^3 \right\} & for\ 0 < h < C_2 \\ \gamma(h) = C_0 + C_1 & for\ h \geq C_2 \end{cases}$$

C_0 is the value of nugget parameter, representing the variability observed at a smaller scale than the minimum sampling interval or the measure errors, while the sill parameter is given by $C_0 + C_1$ that is, the maximum semivariance $\gamma(h)$. C_2 suggests that beyond this distance, the observations are independent of each other and are no longer spatially correlated [8].
We plotted experimental and fitted models using the average LB incidence from 2016 to 2019 and observed spatial autocorrelation in the distribution of LB within 110 km (Figure S1).

Figure S1. Fitted Spherical model (red line) and estimated pairs of points (blue dots).

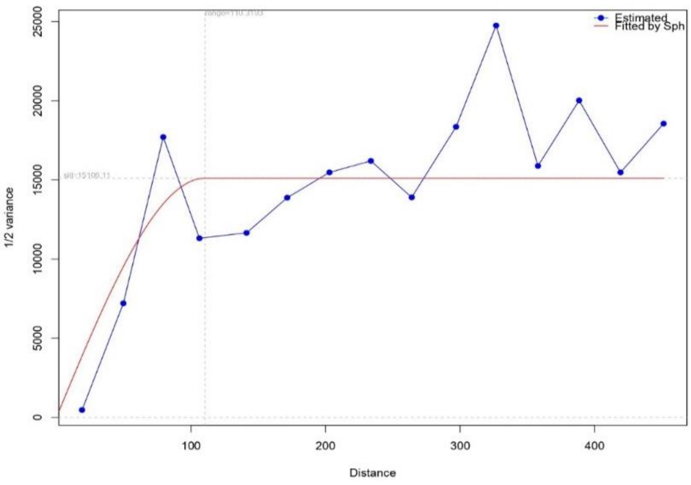

Source 1 The French map with a resolution of approximately 0.2 × 0.2 decimal degrees (dd) used in this study can be download from the website https://github.com/wenfu27/TPBM_LB/tree/main/map in shapefile format.

Figure S2. Smoothed maps of quarterly kriged Lyme borreliosis incidence, France, 2016–2019.

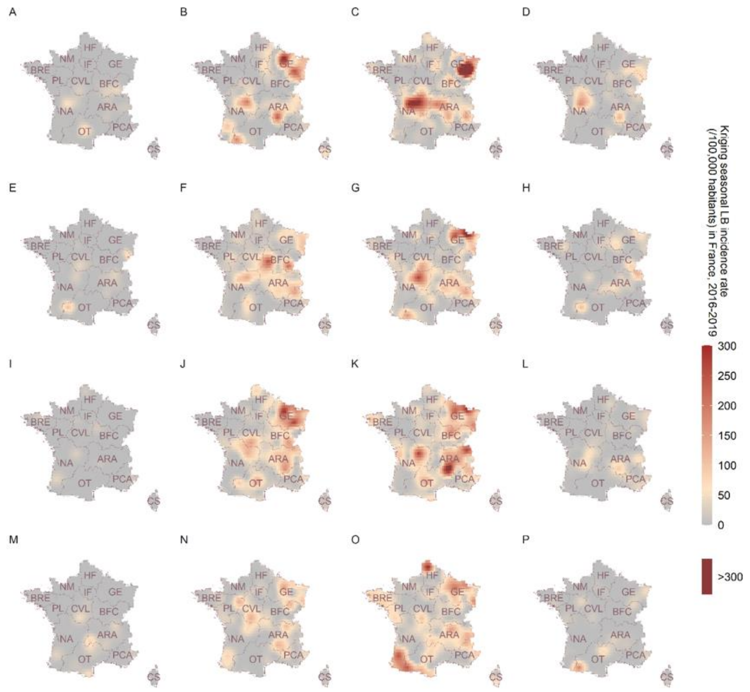

Panels A–D show 2016 kriged values, E–H show 2017 kriged values, I–L show 2018 kriged values and M–P show 2019 kriged values. Each column from left to right indicates the winter (January to March), spring (April to June), summer (July to September) and autumn (October to December) of each year. The darker red areas indicate those with a higher kriged incidence, while grey areas indicate those with low kriged LB incidence. A French shapefile with a resolution of ca 0.2 x 0.2 decimal degrees (~ 22 km^2) was used in this study (See Supplementary Source 1 for the French shapefile template).

Figure S3. Smoothed maps of quarterly kriged Lyme borreliosis incidence, France, 2020–2021.

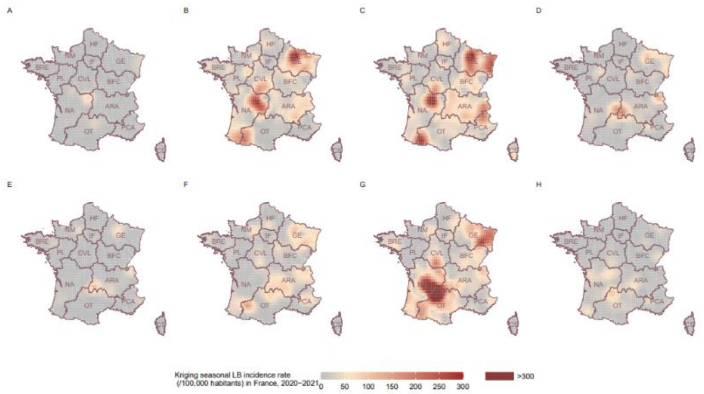

Panels A–D show 2020 kriged values, E–H show 2021 kriged values. Each column indicates in turn the winter (January to March), spring (April to June), summer (July to September), and autumn (October to December) of each year. The darker red areas indicate those with a higher kriged incidence, while grey areas indicate those with low kriged LB incidence.

Each of the 13 regions in mainland France is indicated on the maps by initials (HA: Hauts-de-France; NM: Normandie; IF: Île-de-France; GE: Grand Est; BRE: Bretagne; PL: Pays de la Loire; CVL: Centre-Val de Loire; BFC: Bourgogne-Franche-Comté; NA: Nouvelle Aquitaine; ARA: Auvergne-Rhône-Alpes; OT: Occitanie; PCA: Provence-Alpes-Côte d'Azur; CS: Corse).

Supplementary Material S3: Description of meteorological covariates.

Figure S4. Quarterly averaged daily mean saturation deficit (SD) in France, 2016–2021.

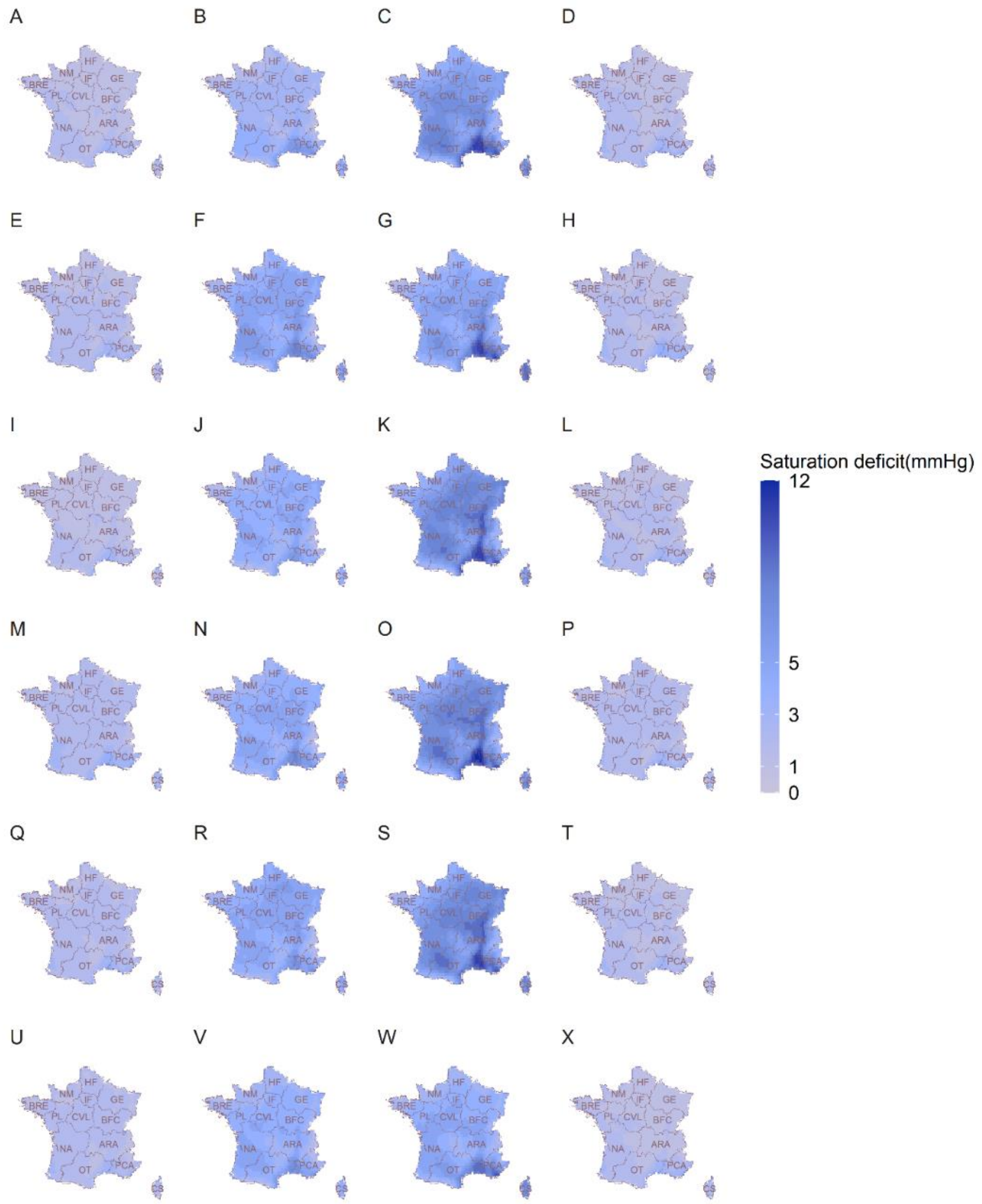

Panels A–D show 2016 SD values, E–H show 2017 SD values, I–L show 2018 SD values, M–P show 2019 SD values, Q–T show 2020 SD values and U–X show 2021 SD values. Each column indicates in turn the winter (January to March), spring (April to June), summer (July to September), and autumn (October to December) of each year. Darker blue indicates areas with higher SD values (drier and warmer areas), while light purple indicates areas with lower SD values (wetter and colder areas). A French shapefile with a resolution of ca 0.2 x 0.2 decimal degrees (~ 22 km2) was used in this study (See Supplementary Source 1 for the French shapefile template).

Each of the 13 regions in mainland France is indicated on the maps by initials (HA: Hauts-de-France; NM: Normandie; IF: Île-de-France; GE: Grand Est; BRE: Bretagne; PL: Pays de la Loire; CVL: Centre-Val de Loire; BFC: Bourgogne-Franche-Comté; NA: Nouvelle Aquitaine; ARA: Auvergne-Rhône-Alpes; OT: Occitanie; PCA: Provence-Alpes-Côte d'Azur; CS: Corse).

Figure S5. Quarterly averaged daily maximum soil temperature (ST) in France, 2016–2021.

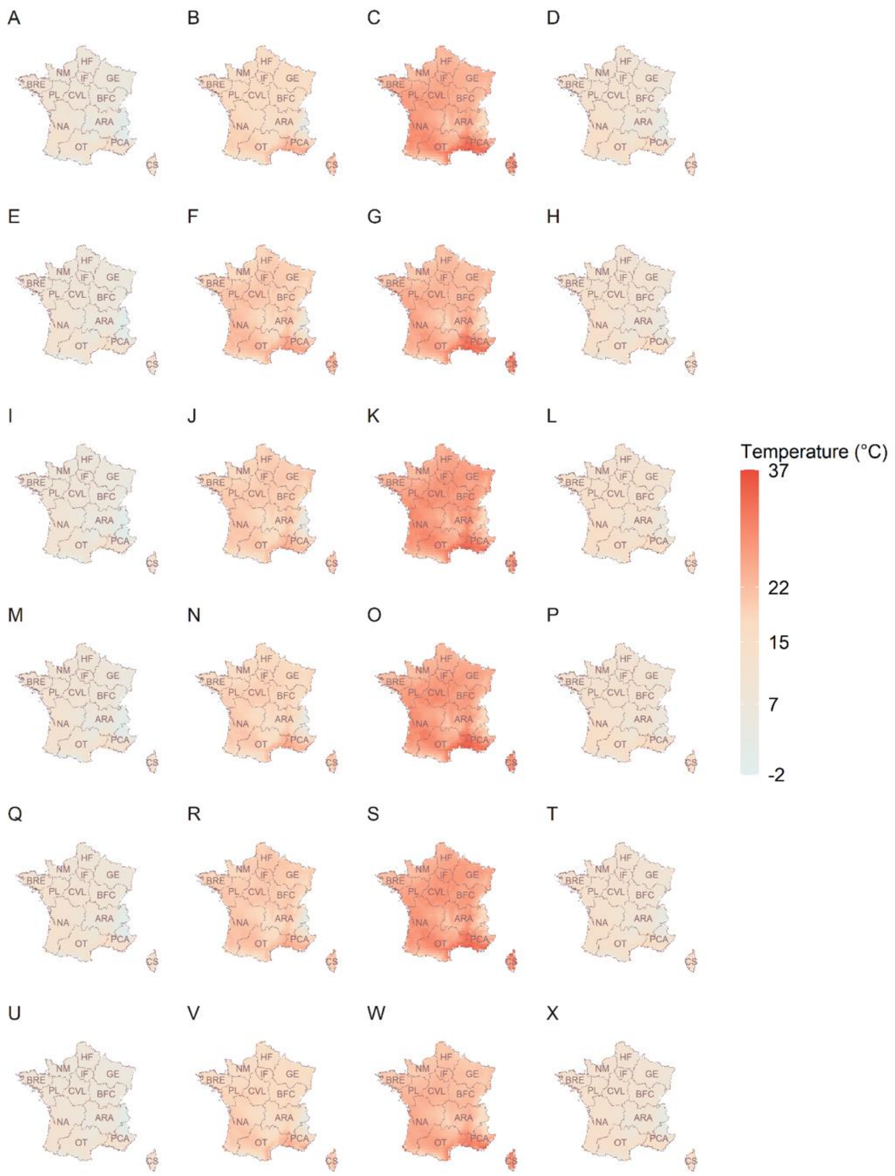

Panels A–D show 2016 ST values, E–H show 2017 ST values, I–L show 2018 ST values, M–P show 2019 ST values, Q–T show 2020 ST values and U–X show 2021 ST values. Each column indicates in turn the winter (January to March), spring (April to June), summer (July to September), and autumn (October to December) of each year. Orange indicates areas with higher soil temperatures, while light color indicates areas with cool soil temperatures. A French shapefile with a resolution of ca 0.2 x 0.2 decimal degrees (~ 22 km2) was used in this study (See Supplementary Source 1 for the French shapefile template).

Each of the 13 regions in mainland France is indicated on the maps by initials (HA: Hauts-de-France; NM: Normandie; IF: Île-de-France; GE: Grand Est; BRE: Bretagne; PL: Pays de la Loire; CVL: Centre-Val de Loire; BFC: Bourgogne-Franche-Comté; NA: Nouvelle Aquitaine; ARA: Auvergne-Rhône-Alpes; OT: Occitanie; PCA: Provence-Alpes-Côte d'Azur; CS: Corse).

Supplementary Material S4: Prediction maps of the logistic model, 2020–2021.

Figure S6. Predicted probability of seasonal Lyme borreliosis presence in France, 2020–2021.

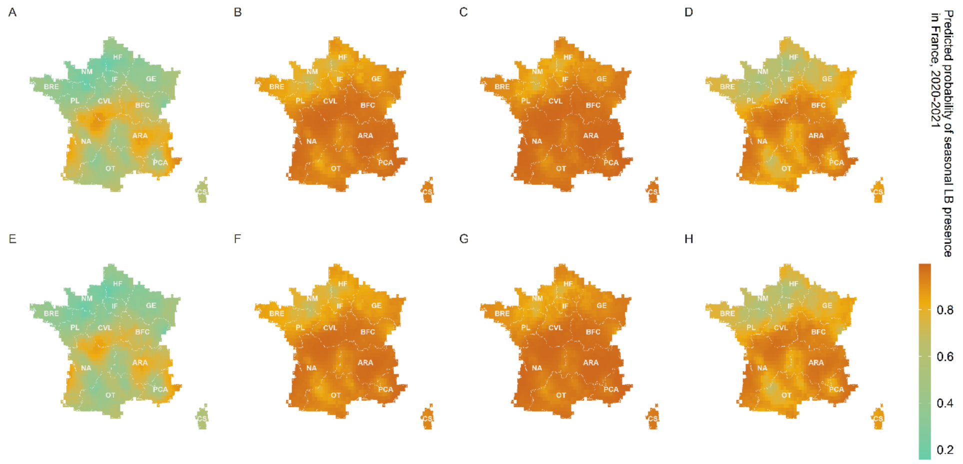

Panels A–D show 2020 predicted probability, E–H show 2021 predicted probability. Each column from left to right indicates the winter (January to March), spring (April to June), summer (July to September) and autumn (October to December) of each year. The darker orange colour indicates a higher probability of LB presence, while the lighter green indicates a lower probability. A French shapefile with a resolution of ca 0.2 x 0.2 decimal degrees (~ 22 km2) was used in this study (See Supplementary Source 1 for the French shapefile template).

Each of the 13 regions in mainland France is indicated on the maps by initials (HA: Hauts-de-France; NM: Normandie; IF: Île-de-France; GE: Grand Est; BRE: Bretagne; PL: Pays de la Loire; CVL: Centre-Val de Loire; BFC: Bourgogne-Franche-Comté; NA: Nouvelle Aquitaine; ARA: Auvergne-Rhône-Alpes; OT: Occitanie; PCA: Provence-Alpes-Côte d'Azur; CS: Corse).

Figure S7. Standard deviation of predicted seasonal incidence rate (/100, 000 inhabitants), France, 2016–2021.

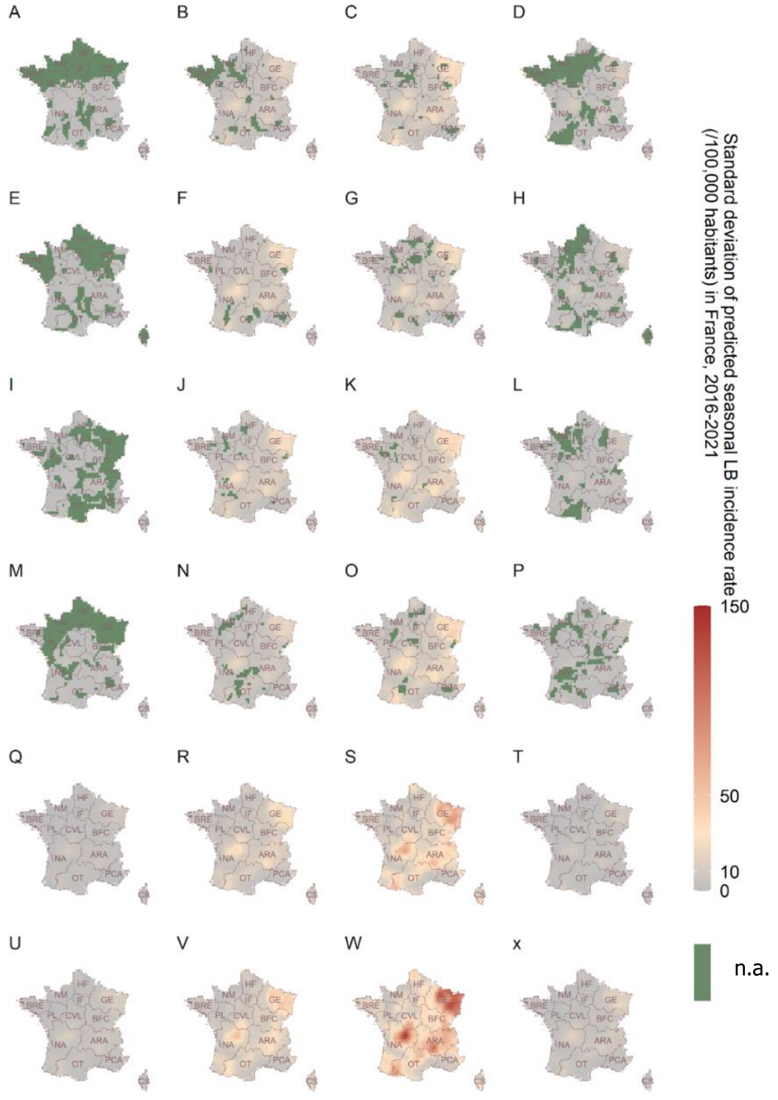

LB: Lyme borreliosis; n.a.: not included in the gamma model.

Panels A–D show 2016 values, E–H show 2017 values, I–L show 2018 values, M–P show 2019 values, Q–T show 2020 values and U–X show 2021 values. Each column indicates in turn the winter (January to March), spring (April to June), summer (July to September), and autumn (October to December) of each year. Dark red color indicates areas with higher variability of prediction, while the green color indicates areas where the kriged incidences are 0 that not included in the gamma model. A French shapefile with a resolution of ca 0.2 x 0.2 decimal degrees (~ 22 km2) was used in this study (See Supplementary Source 1 for the French shapefile template).

Each of the 13 regions in mainland France is indicated on the maps by initials (HA: Hauts-de-France; NM: Normandie; IF: Île-de-France; GE: Grand Est; BRE: Bretagne; PL: Pays de la Loire; CVL: Centre-Val de Loire; BFC: Bourgogne-Franche-Comté; NA: Nouvelle Aquitaine; ARA: Auvergne-Rhône-Alpes; OT: Occitanie; PCA: Provence-Alpes-Côte d'Azur; CS: Corse).

Supplementary Material S5: The other tested multivariable models

Table S1 Results of the tested multivariable models, parameters included and the WAIC values.

	Model description	WAIC
1	NDVI+Rodent species+Deer+ST+SD+Tick bite reports	176219.52
2	NDVI+Rodent species+ST+SD +Rainless days+Tick bite reports	176221.85
3	NDVI+Rodent species+Deer+ST+SD	176224.00
4	NDVI+Rodent species+Deer+ST+SD+Rainless days+Tick bite reports	176240.36
5	NDVI+Deer+SD+Tick bite reports	176247.64
6	NDVI+SD+Rainless days+Tick bite reports	176263.26
7	NDVI+Rodent species+SD+Rainless days+Tick bite reports	176265.82
8	NDVI+Deer+SD+Rainless days+Tick bite reports	176279.75
9	NDVI+Deer+ST+SD	176310.27
10	NDVI+Deer+SD+Rainless days	176315.81
11	NDVI+Rodent species+Deer+SD+Rainless days+Tick bite reports	176319.99
12	NDVI+Deer+SD	176321.79

NDVI: normalised difference vegetation index; SD: saturation deficit; ST: soil temperature.

Supplementary Material S6: Cross-validation of the gamma model

Figure S8. Histogram of the probability integral transform (PIT), 2020.

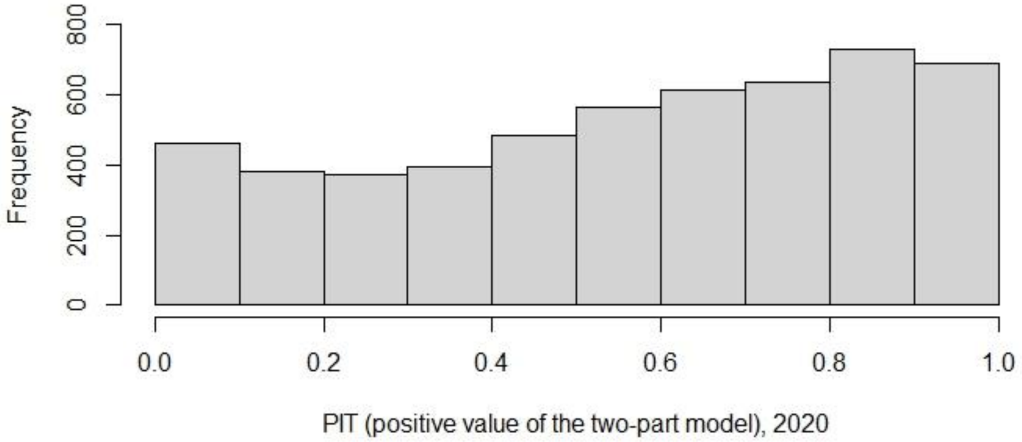

Figure S9. Histogram of the probability integral transform (PIT), 2021.

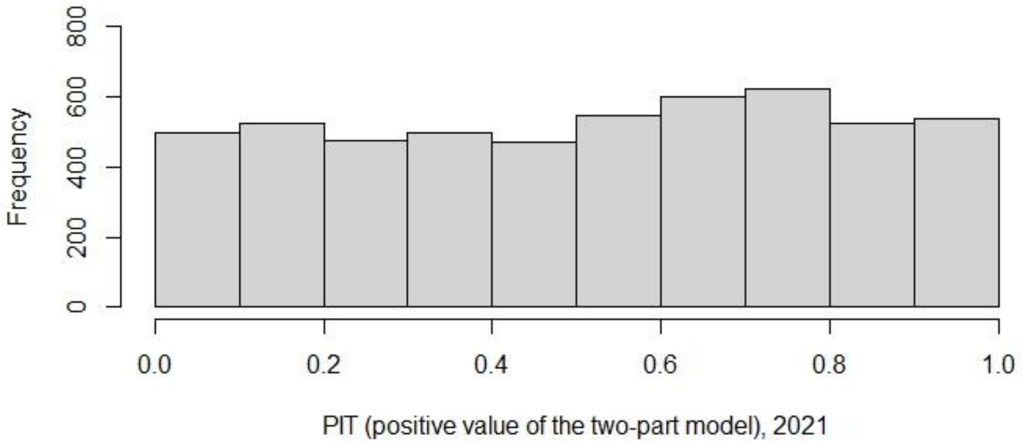

Figure S10. The overall national predicted and observed annual Lyme borreliosis incidence rate per 100,000 inhabitants, France, 2016–2021.

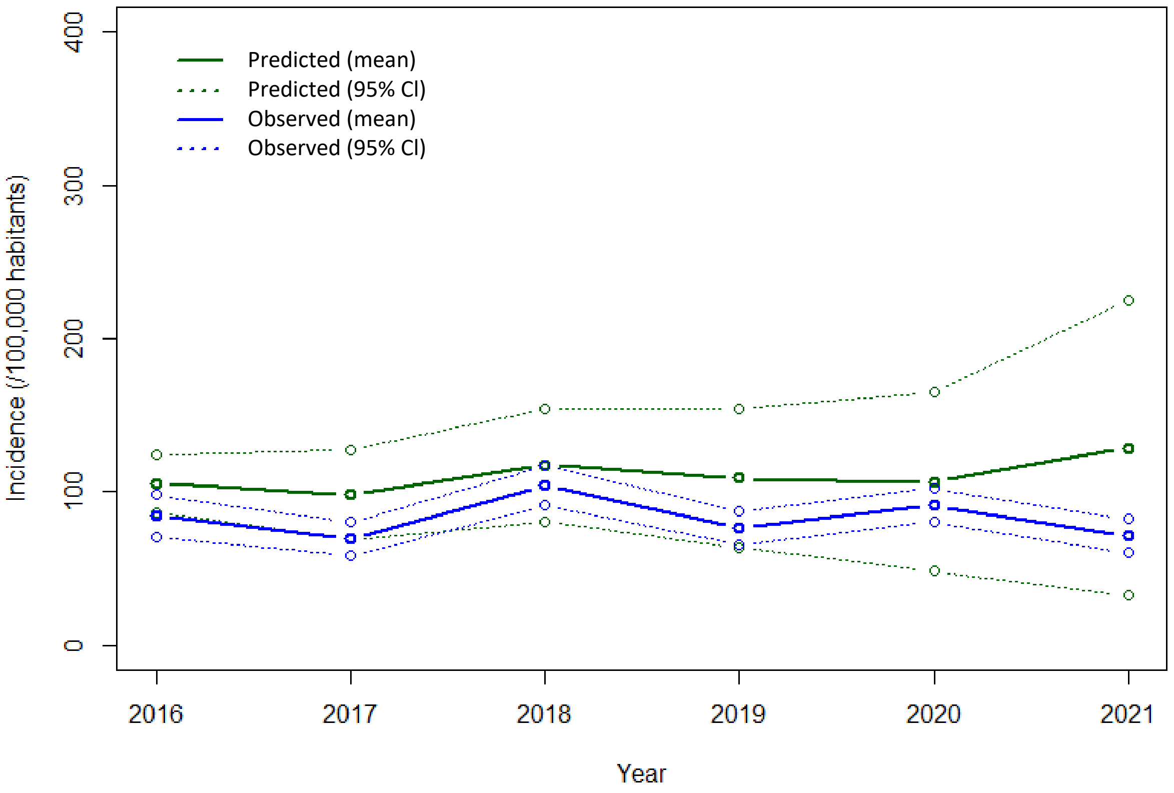

The predicted mean incidence values and their confidence interval (95% CI) were calculated from all grid cells and quarters of the gamma model and for each year (in green). The observed mean incidence values and their 95% CI were those calculated by the Réseau Sentinelles (in blue) [9].

References

1. INRAE - National Research Institute for Agriculture, Food and the Environment. Citique | Citoyen et tiques - un programme de science participative ! Nancy:Programme CiTIQUE. French. [Accessed: 03 Jan 2021]. Available: https://www.citique.fr/
2. Frey-Klett P, Brun-Jacob A, Marchand J, Boniface P, Ortmans C, Salvat G, et al. La recherche participativecitique. Rev For Fr. 2018;70: 205–208. doi:10.4267/2042/69997
3. Copernicus Land Monitoring Service. Corine Land Cover 2018. [Accessed: 01 Jan 2023]. Available: https://land.copernicus.eu/pan-european/corine-land-cover/clc2018
4. R Core Team (2022). R: A language and environment for statistical computing. R Foundation for Statistical Computing, Vienna, Austria. [Accessed: 03 Jan 2021]. Available from: https://www.R-project.org/.
5. Institut national de la statistique et des études économiques (Insee). Historique des populations depuis 1876. [Population history since 1876]. Montrouge: Insee. [Accessed: 01 Jan 2023]. French. Available: https://www.insee.fr/fr/information/2414405
6. Global Administrative Areas. GADM database of French Administrative Areas, version 4.1. [Accessed: 01 Jan 2023]. Available: www.gadm.org.
7. Géoservices | Accéder au téléchargement des données libres IGN. Saint-Mndé: IGN. [Accessed: 01 Jan 2023]. French. Available: https://geoservices.ign.fr/documentation/diffusion/telechargement-donnees-libres.html
8. Kleijnen JPC. Kriging: Methods and Applications.Tilburg: CentER, Center for Economic Research. 2017. doi:10.2139/ssrn.3075151
9. Réseau Sentinelles. Paris: Réseau Sentinelles. French. [Accessed: 01 Jan 2023] Available: https://www.sentiweb.fr/france/fr/?page=maladies&mal=18
